# Reconstruction of large truncal defects post-malignant tumor excision using triple rhomboid flaps

**DOI:** 10.3389/fmed.2026.1871367

**Published:** 2026-07-07

**Authors:** Kai Xie, Yilei Wu, Nan Cao, Xuan Guo, Weixing Xie, Xueqing Wang, Honglei Wang, Zhaojun Yuan, Guangliang Zhang

**Affiliations:** 1 Dermatology Hospital of Shandong First Medical University, Jinan, Shandong, China; 2 Shandong Provincial Institute of Dermatology and Venereology, Shandong Academy of Medical Sciences, Jinan, Shandong, China

**Keywords:** random pattern flap, reconstruction, skin defect, skin malignancies, triple rhomboid flap

## Abstract

**Objective:**

The reconstruction of large defect after skin malignant tumor excision is a challenge in dermatologic surgery and reconstructive surgery. This study describes the use of triple rhomboid flaps for reconstruction of large truncal defects after excision of malignant skin tumors.The research aims to present an accessible and efficacious method for reconstructing large truncal defects using triple rhomboid flaps.

**Methods:**

Between April 2023 and June 2025, nine patients with large skin malignant tumors in the trunk, with dimensions ranging from 5×5 cm to 17×15 cm, were treated at our hospital. We use triple rhomboid flaps to reconstruct the defects.

**Results:**

No major postoperative complications were seen. All flaps survived, and incisions healed in a single phase. Both medical professionals and patients expressed satisfaction with the results.

**Conclusion:**

In this small retrospective case series, the triple rhomboid flap appeared to be a feasible, practical, and easy-to-perform reconstructive strategy for selected large truncal defects after malignant skin tumor excision. Its simple design and straightforward operative procedure may make it a useful option for dermatologic surgeons, particularly in settings where complex microsurgical reconstruction is not routinely available. Further studies with larger cohorts, longer follow-up, and comparative designs are needed to validate its indications, safety, and long-term outcomes.

## Introduction

The reconstruction of large defect after skin malignant tumor excision is a challenge in dermatologic surgery and reconstructive surgery (1). Tumors such as melanoma, dermatofibrosarcoma protuberans, basal cell carcinoma, Bowen disease, and cicatricial carcinoma may require wide local excision to achieve adequate oncologic clearance. On the trunk, these resections can leave sizable circular or oval defects that are difficult to close primarily, particularly when the defect is large, the surrounding skin is under tension, or the surgical site is close to functionally important anatomical regions.

Several reconstructive methods have been reported for large skin and soft-tissue defects, including skin grafting, artificial dermis, pedicled myocutaneous flaps, and free flaps (2). However, each method has specific limitations. Skin grafting is technically simple but may provide limited tissue thickness, relatively poor resistance to friction or mechanical stress, and possible color or texture mismatch. Artificial dermis is useful in selected cases, but its relatively high cost may restrict routine application. More complex flap procedures, especially free-flap reconstruction, can provide robust tissue coverage but require advanced technical expertise and may cause additional donor-site morbidity (3). Therefore, a simple local reconstructive option remains valuable for selected large truncal defects. In this study, we report our experience using triple rhomboid flaps to reconstruct large defects after excision of malignant skin tumors of the trunk.

## Materials and methods

### Patient information and selection criteria

This retrospective case series included nine patients with large skin malignant tumors of the trunk who were treated at the Hospital for Skin Diseases, Shandong First Medical University, between April 2023 and June 2025. The patient cohort included six males and three females with an average age of 58.8 years (range, 20–77 years). The tumors comprised melanoma (*n* = 3), basal cell carcinoma (*n* = 2), Bowen disease (*n* = 1), dermatofibrosarcoma protuberans (*n* = 2) and cicatricial carcinoma (*n* = 1). Defect locations included the intercostal region (four cases), back (four cases), and lateral chest wall (one case).

Patients were considered suitable for triple rhomboid flap reconstruction when the lesion was located on the trunk, complete tumor excision could be achieved without compromising the required oncologic margin or depth, the resulting defect was considered suitable for local fasciocutaneous flap coverage, and the surrounding skin had sufficient laxity for flap mobilization and closure. In this series, the resection depth in some patients with dermatofibrosarcoma protuberans extended below the deep fascia and included partial muscle resection, and triple rhomboid flap reconstruction was still feasible. However, for deeper or more complex defects, other reconstructive options should be selected according to the oncologic and anatomical requirements.

### Surgical technique

**Figure 1:**
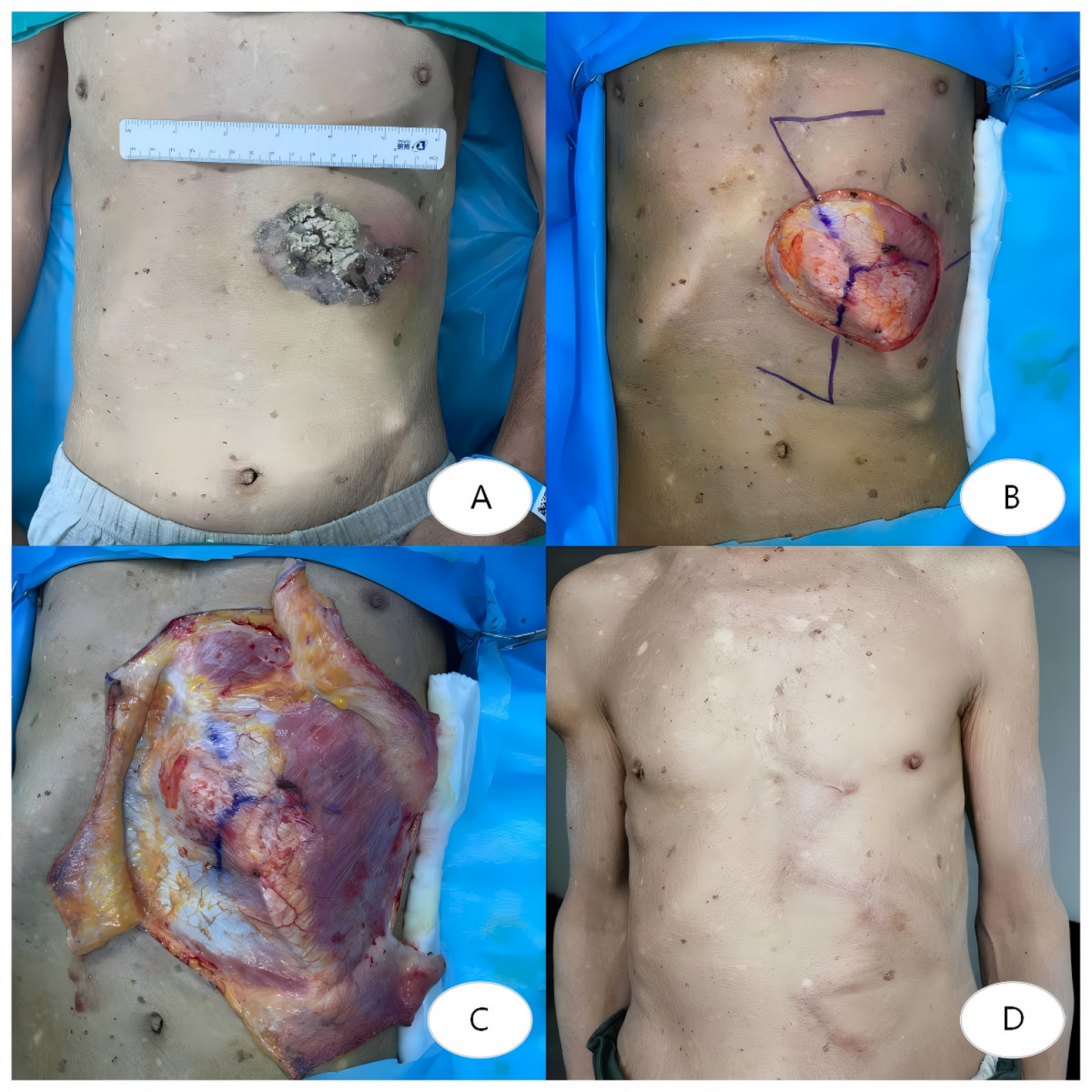
**(A)** Giant squamous cell carcinoma of the trunk. **(B)** Design of the triple rhomboid flaps. **(C)** Performing the flap dissection on the surface of the deep fascia. **(D)** The wound healed well 1 year after surgery.

The operation was performed under general anesthesia. The rhomboid flaps were designed according to the area of the wound after the tumor resection. Markings for the rhomboid flaps were made following Limberg's method, dividing the circular wound into three equal sectors (4) (Figures 1A, B).

**Figure 2:**
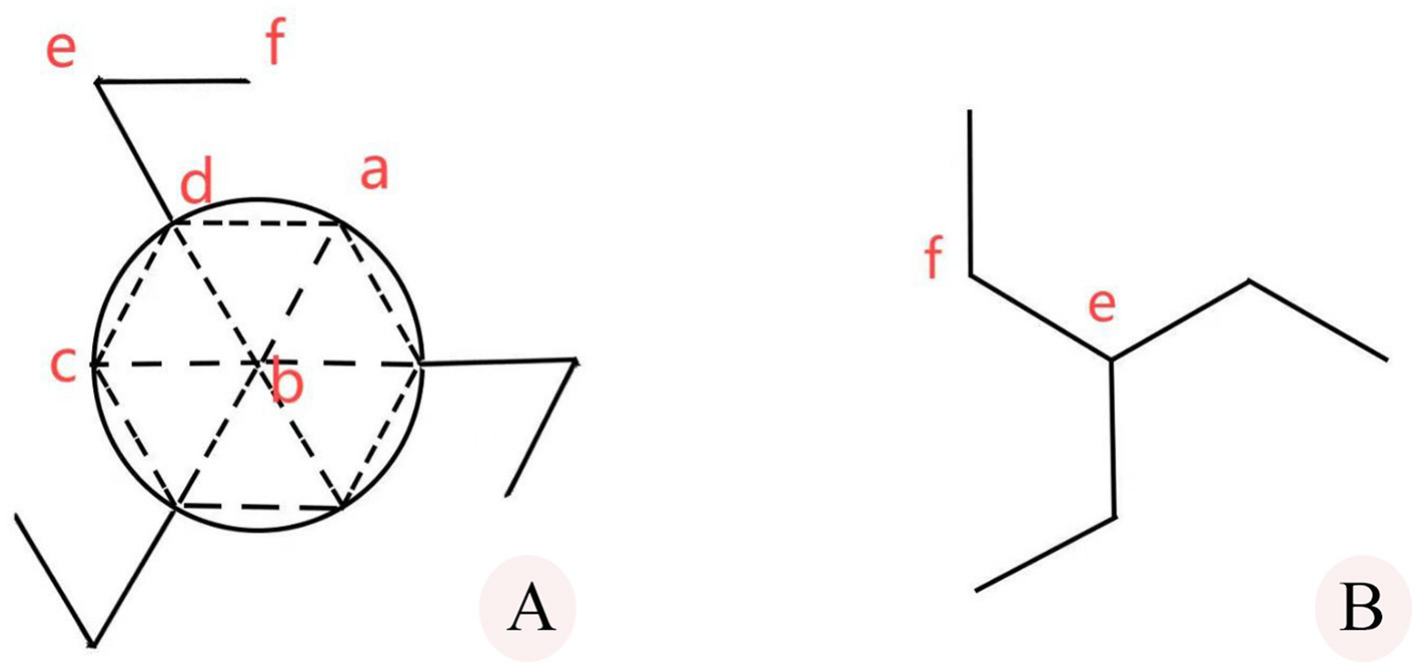
Design of the triple rhomboid flaps. **(A)** Flap design. **(B)** Schematic illustration after flap rotation and closure.

The rhomboid flaps were designed with sides equivalent to the wound's radius. As shown in Figure 2, each side of the rhombus ABCD is equal to the radius of the circular defect. ED is an extension of BD to the same length, EF is equal in length to ED, and the angle < FED is 60°. Each rhombic flap is rotated counterclockwise to be assembled, and the secondary defects are sutured in layers.

Surgical dissection at the deep fascia level prepared the flaps, ensuring the preservation of perforator vessels (Figure 1C). After achieving complete hemostasis and rinsing the wound with normal saline, the flaps were rotated and adjusted to cover the defect. The closure was performed under minimal tension, and drainage tubes were placed under the flaps.

### Postoperative management and outcome assessment

Postoperative wound dressing should be changed every 2 days, and the blood circulation of the flap should be observed. These drainage tubes were removed once the output fell below 10 ml per day. After 2 weeks, sutures were removed. Follow-up duration ranged from 6 to 16 months.

Postoperative outcomes included flap survival, wound healing, wound dehiscence, infection, hematoma, seroma, partial flap necrosis, operative time, drainage duration, patient satisfaction, scar quality, and functional impairment. Patient satisfaction with the reconstructive outcome was assessed using the Visual Analogue Scale (VAS), with higher scores indicating greater satisfaction. Scar quality was assessed using the Vancouver Scar Scale (VSS), with lower scores indicating more favorable scar characteristics. The VAS and VSS evaluations were performed during postoperative follow-up by physicians with comparable clinical experience who were not involved in the operations. Because these evaluations were not blinded, potential assessment bias was still considered in the interpretation of the results.

## Results

The size of the resected area ranged from 5 × 5 cm to 17 × 15 cm, and the size of a single flap varied from 2.5 × 2.5 cm to 8 × 8 cm. The mean operative time was 92.22 ± 15.43 min (range, 75–120 min). Drainage tubes were placed in six patients, and the drainage duration among these patients ranged from 6 to 14 days, with a mean of 8.50 ± 3.56 days. In three patients with smaller defects and limited dead space, drainage tubes were not used. Patient details are outlined in Table 1. After the operations, all flaps exhibited successful survival and healed by primary intention. Every patient underwent a thorough evaluation during the initial monthly follow-up visit. The outcomes were deemed satisfactory, with no reported functional impairments (Figure 1D). All 9 patients underwent postoperative assessment: mean VAS score was 8.33 ± 0.71 (7–9; 88.9% ≥8), showing high satisfaction with repair; mean VSS score was 2.22 ± 0.97 (1–4; 66.7% ≤ 2), indicating favorable scar healing and satisfactory aesthetic results. Favorable therapeutic outcomes were achieved in all patients, with overall satisfactory scale scores (Supplementary Figure 1).

**Table 1:** The main information of each patient.

No	Gender	Age (year)	Diagnosis	Size of defect (cm × cm)	Size of flap (cm × cm)	Complication	VAS	VSS
1	Female	67	melanoma	8 × 8	4 × 4	None	8	2
2	Male	58	basal cell carcinoma	17 × 15	8 × 8	None	7	4
3	Male	67	Bowen disease	10 × 10	5 × 5	None	8	2
4	Male	38	Dermatofibrosarcoma protuberans	12 × 13	6 × 6	None	8	3
5	Female	61	Melanoma	7 × 7	3.5 × 3.5	None	9	2
6	Male	71	Basal cell carcinoma	7 × 6	3 × 3	None	8	2
7	Male	70	Melanoma	5 × 5	2.5 × 2.5	None	9	1
8	Male	77	Cicatricial carcinoma	5 × 5	2.5 × 2.5	None	9	1
9	Female	20	Dermatofibrosarcoma protuberans	6 × 6	3 × 3	None	9	3

## Discussion

Reconstructing large defects following the excision of skin malignant tumors poses a significant challenge in both dermatologic and reconstructive surgery (5). Several reconstructive methods have been used for such defects, including skin grafting, artificial dermis, local flaps, musculocutaneous flaps, perforator flaps, and free flaps (6). However, each method has specific limitations. Skin grafting is technically simple and widely available, but the grafted skin may provide limited tissue thickness and relatively poor resistance to friction or mechanical stress, especially in areas exposed to repeated movement or pressure. It may also result in color or texture mismatch. Artificial dermis can be useful in selected wounds, but its relatively high cost and possible need for staged reconstruction may restrict its routine use. Musculocutaneous, perforator, and free flaps can provide robust tissue coverage for complex defects, but these procedures are usually more technically demanding. In particular, free-flap reconstruction requires microsurgical expertise, vascular anastomosis, longer operative preparation, and may be associated with donor-site morbidity.

The rhomboid flap is a type of random pattern flap. First proposed by Limberg in 1946 (7), it's a series of communicating equilateral triangles. All angles are 60°, which means that each side of both the defect and the flap is equal in length. Its geometric design is simple and reproducible, and it has been widely used for reconstruction of cutaneous defects in different anatomical regions. For larger defects, the design can be modified into a triple rhomboid flap by dividing a circular or nearly circular defect into three equal sectors and rotating three rhomboid flaps to close the primary defect. Through extensive dissection at the deep fascial level, direct suture at the secondary defect area can enhance flap mobility, reducing scar tissue proliferation. This method may provide a practical option for large defects after tumor excision.

In previous literature, the triple rhomboid flap has been reported to be used for repairing smaller defects on the scalp or neonatal meningomyelocele defects (4). Its application in adult trunk large-area defect cases remains rare. In the present case series, all nine patients underwent reconstruction with triple rhomboid flaps after excision of large truncal malignant skin tumors. The defects ranged from 5 × 5 cm to 17 × 15 cm, and all flaps survived without major postoperative complications. No wound dehiscence, infection, hematoma, seroma, or partial flap necrosis was observed. The postoperative VAS satisfaction scores and VSS scar scores suggested satisfactory short- to mid-term cosmetic outcomes. The recorded operative time and drainage duration provided additional objective perioperative information and supported the technical feasibility of this approach in selected patients. Nevertheless, because this study lacked a control group, these results should be interpreted cautiously as preliminary observations rather than evidence that this technique is superior to other reconstructive methods.

As a local fasciocutaneous flap, the triple rhomboid flap has several practical advantages in selected truncal defects. It uses adjacent tissue with similar texture and thickness, allows primary closure with less tension, and avoids vascular anastomosis or sacrifice of major muscle tissue. The design is easy to understand and reproduce, which may be useful for dermatologic surgeons in centers where microsurgical reconstruction is not routinely performed.

Nevertheless, the indications of this technique should be carefully selected. Oncologic safety must always take priority over flap design. In the present series, some dermatofibrosarcoma protuberans cases required resection beyond the deep fascia or removal of part of the underlying muscle, and the resulting defects could still be repaired using triple rhomboid flaps. Therefore, the use of this flap did not restrict the necessary depth of tumor excision in these selected cases. However, for defects involving more extensive deep tissue loss or exposure of important structures, adequate oncologic resection should not be compromised to permit local flap closure. In such cases, other reconstructive options, such as musculocutaneous flaps, pedicled flaps, perforator flaps, free flaps, or staged reconstruction, may be more appropriate. Therefore, the triple rhomboid flap is best considered for selected truncal defects in which complete tumor excision can be achieved and surrounding skin laxity is sufficient for local flap transfer.

The present study has several limitations. First, it was a small retrospective case series with only nine patients from a single institution. Second, no control or comparison group was included, so direct comparison with skin grafting, artificial dermis, perforator flaps, or free flaps was not possible. Third, the patient cohort was heterogeneous, including different tumor types, defect sizes, and anatomical locations, which may influence surgical difficulty, closure tension, scar formation, and oncologic outcomes. Fourth, the follow-up period ranged from 6 to 16 months, which may be sufficient to assess early wound healing and short- to mid-term scar appearance, but is not long enough to fully evaluate long-term scar maturation, functional outcomes, or tumor recurrence. Fifth, although VAS and VSS were assessed by physicians who were not involved in the operations, these scores remain partly subjective. In addition, hospital stay was not included as a comparative variable because it may be influenced by tumor type, oncologic evaluation, postoperative observation requirements, and individual patient conditions rather than the reconstructive method alone.

In conclusion, the triple rhomboid flap was a feasible reconstructive option for selected large truncal defects after malignant skin tumor excision in this small retrospective case series. Its geometric design is simple and the procedure is easy to reproduce, which may be useful for dermatologic surgeons in centers where microsurgical reconstruction is not routinely performed. Larger comparative studies with longer follow-up are needed to confirm its indications, safety, and long-term outcomes.

## Data Availability

The data that support the findings of this study are available from the corresponding author upon reasonable request.
