# Supplementary material for: Reconstruction of large truncal defects post-malignant tumor excision using triple rhomboid flaps
Source: Front Med (Lausanne). 2026 Jul 7;13:1871367. doi: 10.3389/fmed.2026.1871367 (PMC13384816; doi:10.3389/fmed.2026.1871367)
Supplement: Supplementary file 1 [file Supplementary_file_1.docx]

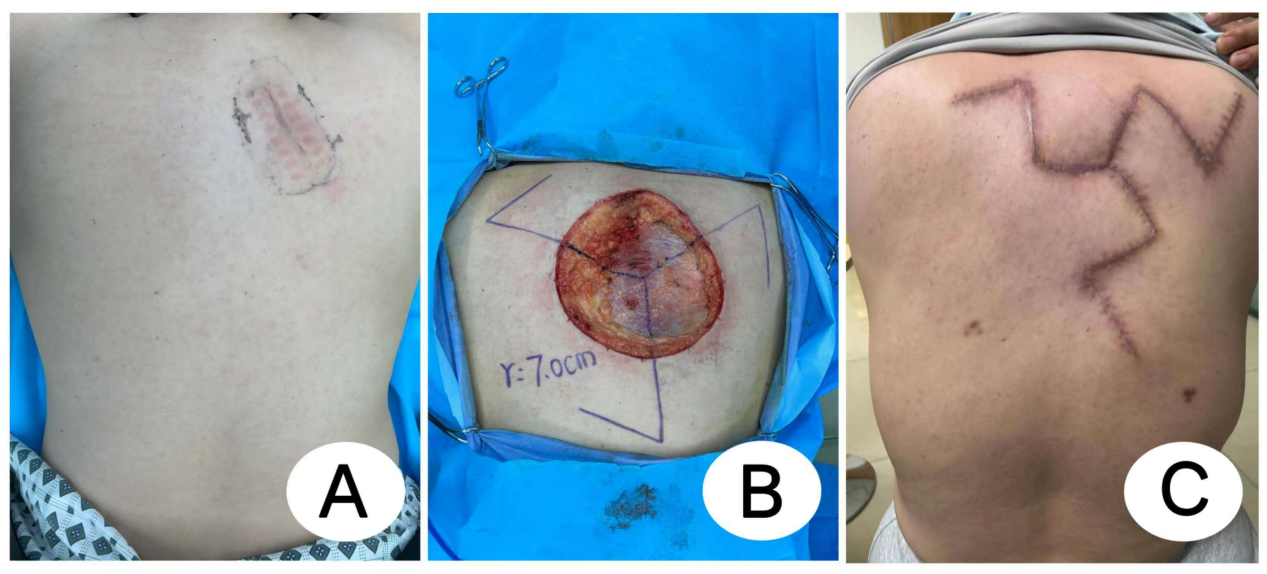


Supplementary Figure 1. Additional representative case treated with triple rhomboid flap reconstruction. (A) Preoperative appearance. (B) Flap design after tumor excision. (C) Postoperative appearance after wound closure.
